# Comprehensive Analysis of Sinonasal Inverted Papilloma Expression Profiles Identifies Long Non-Coding RNA AKTIP as a Potential Biomarker

**DOI:** 10.3389/fgene.2022.831759

**Published:** 2022-02-02

**Authors:** Hanyi He, Xinlu Wang, Yueyue Lu, Xiaojiang Lin, Yuandong Li, Yong Li, Zhihong Lin, Zhiqi Ma, Xiaolin Cao, Yaoshu Teng

**Affiliations:** ^1^ Department of Otorhinolaryngology, Affiliated Hangzhou First People’s Hospital, Zhejiang University School of Medicine, Hangzhou, China; ^2^ The Fourth Clinical Medical College, Zhejiang Chinese Medical University, Hangzhou, China; ^3^ Department of Otorhinolaryngology, Kaihua People’s Hospital, Quzhou, China; ^4^ Department of Otorhinolaryngology, Hangzhou Children’s Hospital, Hangzhou, China; ^5^ Department of Otorhinolaryngology, Second Affiliated Hospital, School of Medicine, Zhejiang University, Hangzhou, China

**Keywords:** inverted papilloma, long noncoding RNA, expression profiles, bioinformatics, biomarker

## Abstract

Long noncoding RNAs (lncRNAs) are a novel class of potential biomarkers and therapeutic targets for the treatment of neoplasms. The purpose of this study was to explore the expression profile, potential functions, and diagnostic and clinical significance of lncRNAs in sinonasal inverted papilloma (SNIP). The expression profiles of lncRNAs and mRNAs were analyzed using a microarray. The potential functions and clinical implications of specific lncRNAs were further analyzed by bioinformatics and statistical methods. Microarray analysis identified 1,668 significantly upregulated and 1,767 downregulated lncRNAs in SNIP. Several mRNAs coexpressed with lncRNAs were enriched in some biological processes and cellular signaling pathways related to tumorigenesis. Lnc-AKTIP might interact with a variety of tumor-associated proteins and transcription factors, such as PCBP2, IRF-1, and p53. Receiver operating characteristic curve analysis for lnc-AKTIP showed an area under the curve of 0.939. Notably, its expression level was significantly decreased in SNIP tissues versus normal tissues and was associated with SNIP staging. Lnc-AKTIP may serve as a valuable diagnostic biomarker and a therapeutic target for SNIP.

## Introduction

Sinonasal inverted papilloma (SNIP) is a challenging benign tumor arising from the Schneiderian mucosa of the nasal cavity and paranasal sinuses, accounting for approximately 0.5–4% of all sinonasal neoplasms (Vrabec, 1994). It has the biological characteristics of local invasiveness, a high recurrence rate and malignant potential (Krouse, 2000). Malignant transformation has been found in 5–15% of inverted papilloma lesions (Govindaraj and Wang, 2014). Human papillomavirus (HPV) is considered to be closely related to the pathogenesis of SNIP. The other considered risk factors for SNIP development include inflammatory infiltration, welding fumes and organic solvents (Zydroń et al., 2018; Fulla et al., 2020; Papagiannopoulos et al., 2020). However, little is known about the underlying molecular genetic alterations, specific pathologic mechanism and diagnostic biomarkers of this clinical entity.

Long noncoding RNAs (lncRNAs) are defined as noncoding RNAs (ncRNAs) that are greater than 200 nucleotides in length (Ranjbar et al., 2021). Compared with other ncRNAs (e.g., miRNAs), lncRNAs have a longer primary structure, can integrate with DNAs and RNAs, and can form a complex and diverse secondary spatial structure to interact with proteins (Bracht et al., 2017). Although lncRNAs do not encode proteins, some studies have shown that lncRNAs play crucial roles in governing a wide range of fundamental biological processes, including genomic imprinting, chromosome inactivation, differentiation and carcinogenesis, at both the transcriptional and post-transcriptional levels. Aberrant expression of lncRNAs is associated with several human diseases, such as various types of tumors, cardiovascular diseases, and neurological diseases (Santoro et al., 2020; Ranjbar et al., 2021). Moreover, these abnormal lncRNAs are also found in circulating blood and/or urine (Brunner et al., 2012; Shi et al., 2016). LncRNAs are a novel class of potential biomarkers and therapeutic targets for the treatment of neoplasms (Khandelwal et al., 2015; Chandra and Nandan, 2017; Wu and Du, 2017).

In our study, differentially expressed lncRNAs and mRNAs were comprehensively identified by detecting lncRNA and mRNA profiles in SNIP tissues. Functional enrichment analysis of mRNAs coexpressed with these lncRNAs was performed *via* bioinformatics methods. These findings were combined with the clinical features of patients with SNIP to explore the clinical significance of specific lncRNAs in SNIP. This study aimed to provide novel information for further research on the pathogenesis of SNIP and to identify candidate diagnostic biomarkers and therapeutic targets.

## Materials and Methods

### Patients and Sample Collection

SNIP tissue samples were obtained from 41 patients with SNIP (29 males and 12 females; mean age 57.6 years; range: 32–85 years). The diagnosis of SNIP was confirmed by histopathological examination. The clinical characteristics (age, gender, smoking status, tumor staging and recurrence) of each SNIP patient were recorded (Table 1). A total of 12 patients with only nasal septum deviation were selected to provide nasal mucosal tissue samples (nine males and three females; mean age 51.2 years; range: 28–64 years as the control group). Patients were admitted to the Department of Otolaryngology, Affiliated Hangzhou First People’s Hospital, Zhejiang University School of Medicine and Department of Otolaryngology, Second Affiliated Hospital, School of Medicine, Zhejiang University between 2012 and 2020. All tissue samples were immediately preserved in RNA*later* Solution (Ambion, TX, United States) within 15 min after resection and then stored at −20°C until use.

**TABLE 1:** Characteristics of 41 patients with SNIP.

Characteristic	N
Age	
<50 years	14
≥50 years	27
Gender	
Male	29
Female	12
Smoking status	
Yes	26
No	15
Tumor staging^a^	
Ⅰ	8
Ⅱ	20
Ⅲ	11
Ⅳ	2
Ⅰ+Ⅱ	28
Ⅲ+Ⅳ	13
Recurrence	
Yes	6
No	35

a Tumor staging is defined according to a staging system for inverted papilloma described by Krouse (2000).

### RNA Extraction

Total RNA was isolated from nasal mucosal tissues using TRIzol Reagent (Life Technologies, Carlsbad, CA, United States) following the manufacturer’s instructions and then quantified using a NanoDrop ND-1000 spectrophotometer (Thermo Fisher Scientific, Waltham, MA). RNA integrity was inspected by an Agilent Bioanalyzer 2100 (Agilent Technologies, Santa Clara, CA, United States), and RNA samples with RNA integrity number (RIN) values ≥ 6.0 and 28S/18S values > 0.7 were deemed acceptable for microarray and reverse transcription (RT) experiments.

### Microarray Assay

A human lncRNA microarray (4 × 180K; v 6.0) was manufactured at the Shanghai Biochip Co., Ltd. (Shanghai, China), which contains 95,956 capture probes for 77,103 lncRNAs and 18,853 RNAs based on the most authoritative databases, such as GENCODE v21, Ensembl, LNCipedia v3.1, Lncrnadb, Noncode v4 and UCSC. Microarray assays were performed according to the manufacturer’s instructions. Briefly, total RNA was amplified and labeled by the Low Input Quick Amp WT Labeling Kit (Agilent Technologies, Santa Clara, CA, United States). Labeled cRNA was purified with the RNeasy mini kit (QIAGEN, GmBH, Germany). Each slide was hybridized with 1.65 μg of Cy3-labeled cRNA using a Gene Expression Hybridization Kit (Agilent Technologies, Santa Clara, CA, United States). After 17 h of hybridization, the slides were washed with the Gene Expression Wash Buffer Kit (Agilent Technologies, Santa Clara, CA, United States). These slides were scanned by an Agilent Microarray Scanner (Agilent Technologies, Santa Clara, CA, United States). Data were extracted with Feature Extraction Software 10.7 (Agilent Technologies, Santa Clara, CA, United States). Raw data were normalized by the quantile algorithm, Gene Spring Software 11.0 (Agilent Technologies, Santa Clara, CA, United States). Differentially expressed lncRNAs and mRNAs with statistical significance between the two groups were identified through volcano plot filtering (fold change ≥2.0 and *p* < 0.05). The Gene Cluster (v 3.0) and Java TreeView software programs were used to perform hierarchical cluster analysis of these differentially expressed lncRNAs and mRNAs.

### Quantitative Real-Time Reverse Transcription PCR

Total RNA was reverse transcribed to cDNA using Prime Script RT Master Mix (TaKaRa, Dalian, China) following the manufacturer’s protocols. qRT-PCR was performed by using SYBR Premix Ex Taq II (TaKaRa, Dalian, China) on the 7900 HT Sequence Detection System (ABI, United States). The primer sequences are listed in Supplementary Tables S1 and were synthesized by Invitrogen (Shanghai, China). Glyceraldehyde 3-phosphate dehydrogenase (GAPDH) was used as an endogenous control, and gene expression was compared by the threshold cycle (2^−ΔΔCt^) method.

### Construction of the LncRNA-mRNA Coexpression Network

Pearson correlation coefficient (PCC) R values were calculated to evaluate the correlation between the differentially expressed lncRNAs and mRNAs. Fisher’s exact test implemented in the cor. test function of R was adopted to estimate the *p* value for each correlation pair. The *p* value was further adjusted to the false discovery rate (FDR) by Bonferroni multiple test correction. The lncRNA-mRNA correlations with an R value ≥ 0.8 and FDR <0.005 were considered statistically significant correlated pairs. The coexpression network showing the significant pairs was visualized by using Cytoscape software (The Cytoscape Consortium, San Diego, CA, United States).

### Gene Function Analysis

The coexpressed mRNAs were imported into the database for Annotation, Visualization, and Integrated Discovery (DAVID) v6.8 (http://david.ncifcrf.gov), which utilized Gene Ontology (GO) and pathway analysis to identify the enriched GO themes and cell signaling pathways of these coexpressed mRNAs. The thresholds were set as *p* < 0.05 and FDR<0.05.

### Bioinformatics Analysis of Specific LncRNAs and mRNAs

The genomic locations of candidate lncRNAs were confirmed by using UCSC Genome Browser (http://genome-asia.ucsc.edu/index.html). Secondary structures were shown *via* RNAfold minimum free energy estimations based on the RNAfold web server (http://rna.tbi.univie.ac.at/cgi-bin/RNAWebSuite/RNAfold.cgi). *cat*RAPID analysis (http://service.tartaglialab.com/) was performed to predict the potential interacting proteins of lncRNAs. Moreover, TRANSFAC (http://www.gene-regulation.com/index2.html) was used to predict the potential transcription factors of lncRNAs.

### Statistical Analysis

Statistical analyses were performed using SPSS software for Windows (version 16.0; SPSS, Inc., Chicago, IL, United States). The differences between the two groups were determined using a two-tailed Student’s *t*-test. Pearson correlation analysis was performed to investigate the linear relationship between the microarray data and qRT-PCR results. A receiver operating characteristic (ROC) curve was established to evaluate the diagnostic accuracy of lncRNAs as biomarkers of SNIP. The chi-square test was used to determine the relationships between clinical characteristics and altered lncRNA expression. *p* < 0.05 was considered statistically significant.

## Results

### Overview of the Expression Profiles of LncRNAs and mRNAs in SNIP Tissues

**FIGURE 1:**
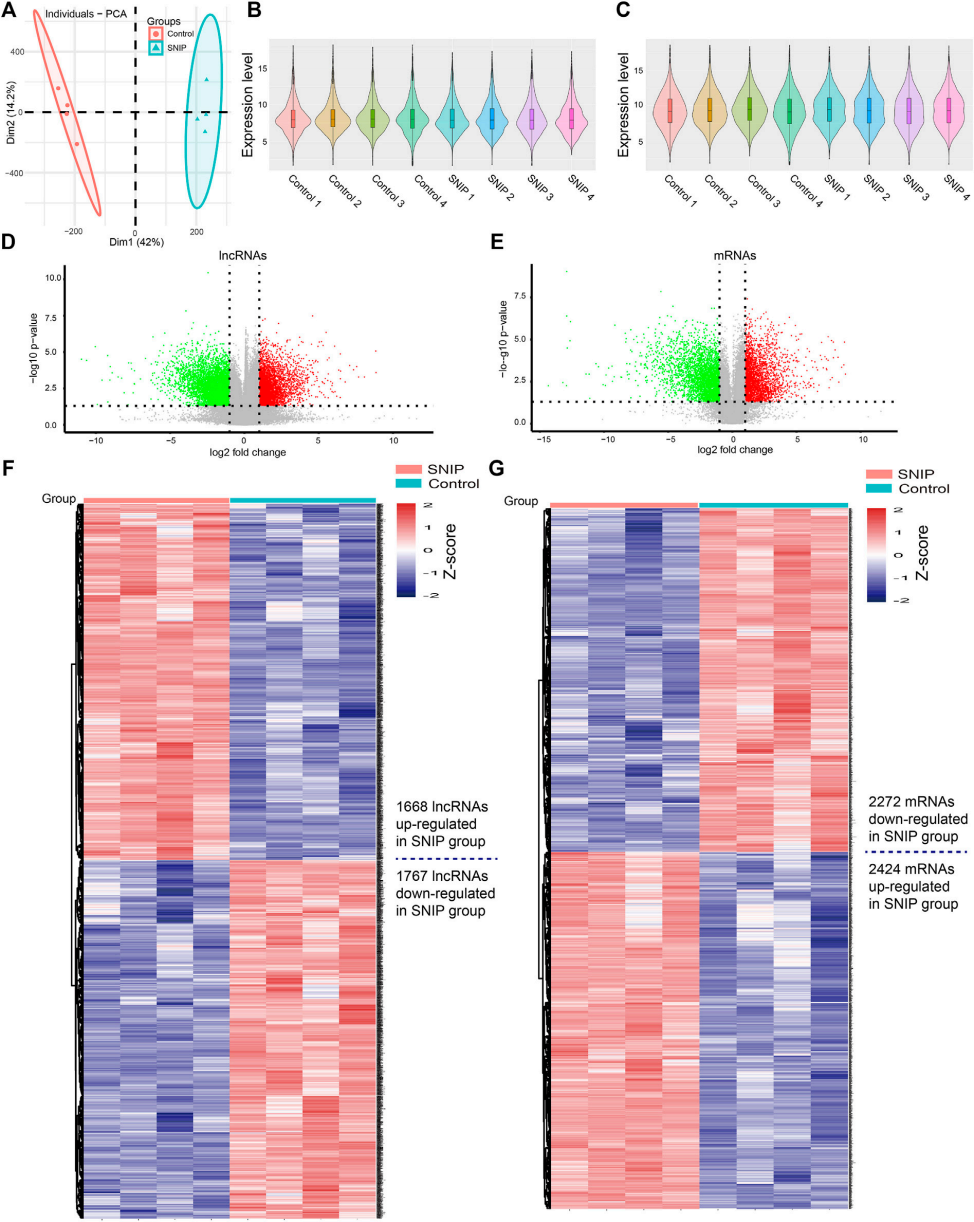
Alterations in lncRNA and mRNA expression profiles between SNIP tissues and nontumorous tissues **(A)** Principal component analysis (PCA) **(B-C)** The violin plot is a convenient way to quickly visualize the distributions of a dataset of lncRNA **(B)** and mRNA **(C)** profiles **(D–E)** The volcano plots show differentially expressed lncRNAs **(D)** and mRNAs **(E)** in the SNIP tissues relative to those from the Control group. The horizontal line represents a *p* value of 0.05 and the vertical lines correspond to 2.0-fold up and down **(F–G)** The heat maps indicate hierarchical clustering results of differentially expressed lncRNAs **(F)** and mRNAs **(G)** (fold change>2; *p* < 0.05). Each row indicates one lncRNA or mRNA, and each column indicates one sample. The lncRNA and mRNA expression levels are illustrated using histograms and Z-scores. The red and blue bars denote high and low relative expression, respectively.

Principal component analysis (PCA) showed that there was a large difference between SNIP tissues and nontumorous tissues (Figure 1A). To identify the differentially expressed lncRNAs and mRNAs potentially involved in SNIP, we first examined the expression patterns of lncRNAs and mRNAs in the SNIP tissues using a microarray assay. Our data showed that a total of 3,435 lncRNAs (1,668 upregulated and 1,767 downregulated) and 4,696 mRNAs (2,424 upregulated and 2,272 downregulated) were significantly differentially expressed in four SNIP tissue samples compared with four corresponding nontumorous tissue samples from the control group (fold change>2; *p* < 0.05) (Figure 1, Supplementary Tables S2,S3). The top 20 differentially expressed lncRNAs and mRNAs are listed in Tables 2, 3. Among these lncRNAs, lnc-SPRR1B-1:1 (log_2_fold change: 8.074459) and lnc-PRH1-1:13 (log_2_fold change: 8.09616) were the most upregulated and downregulated lncRNAs, respectively. In addition, A2ML1 (log_2_fold change: 8.517957) and DMBT1 (log_2_fold change: 12.915) were the most upregulated and downregulated mRNAs, respectively. Our results indicate that these four aberrantly expressed RNAs may play critical roles in the development and progression of SNIP.

**TABLE 2:** Top 20 differentially expressed lncRNAs in SNIP tissues detected by microarray assay.

Upregulated lncRNAs				Downregulated lncRNAs			
lncRNA	Chromosome	log_2_ (fold change)	*p* Value	LncRNA	Chromosome	log_2_ (fold change)	*p* Value
lnc-SPRR1B-1:1	chr1	8.074459	0.000516	lnc-PRH1-1:13	chr12	−8.09616	0.006248
NR_003062	chr1	7.344342	0.000166	lnc-HTN3-2:2	chr4	−7.41738	0.001478
ENST00000411759	chr17	6.782256	0.001133	ENST00000414404	chr2	−6.66018	2.70E-05
lnc-GNRHR-5:2	chr4	6.097429	0.0053	NR_036489	chr18	−5.97944	4.70E-05
NR_126404	chr2	5.899363	0.005206	lnc-ZKSCAN1-3:2	chr7	−5.77709	0.000537
lnc-PROM2-1:2	chr2	5.808771	0.001056	NR_027622	chr3	−5.68696	8.08E-06
NR_073414	chr17	5.606244	0.000893	lnc-RP11-497E19.2.1-3:3	chr14	−5.523	0.00147
NR_104048	chr4	5.282333	0.001278	ENST00000624094	chr11	−5.48935	1.14E-05
NR_073414	chr17	5.234484	0.001084	lnc-BHLHA15-1:1	chr7	−5.37011	0.000427
NR_027054	chr9	5.038543	2.05E-06	lnc-LRRC10-1:2	chr12	−5.08804	6.40E-05
lnc-IL20RB-2:1	chr3	5.021085	0.002429	ENST00000623553	chr16	−5.02176	8.71E-06
NR_120497	chr10	4.946139	0.00024	lnc-MYL9-1:1	chr20	−5.00485	0.000938
lnc-SERPINB3-4:1	chr18	4.919234	0.01212	lnc-GMPPA-2:1	chr2	−5.00169	0.000102
lnc-APLP2-5:2	chr11	4.889049	0.000642	lnc-ANKRD22-1:2	chr10	−4.91236	0.0004
ENST00000420269	chr22	4.862116	0.000494	ENST00000612804	chr19	−4.85893	0.019692
ENST00000596379	chr19	4.839326	0.000487	lnc-RP11-497E19.2.1-3:4	chr14	−4.85061	0.00182
NR_027054	chr9	4.814526	3.70E-05	ENST00000602964	chr5	−4.84958	0.000416
NR_125989	chr1	4.749302	0.00103	lnc-LUC7L-2:1	chr16	−4.74143	0.010327
ENST00000504297	chr5	4.692076	0.001742	lnc-CXCL12-4:1	chr10	−4.69443	5.81E-05
ENST00000566876	chr16	4.588224	3.87E-05	lnc-PTPLB-3:2	chr3	−4.66377	2.11E-05

**TABLE 3:** Top 20 differentially expressed mRNAs in SNIP tissues detected by microarray assay.

Upregulated mRNAs				Downregulated mRNAs			
mRNAs	Chromosome	log_2_ (fold change)	*p* Value	mRNAs	Chromosome	log_2_ (fold change)	*p* Value
A2ML1	chr12	8.517957	0.00045	DMBT1	chr10	−12.915	9.21E-10
KRT6A	chr12	8.29795	0.00339	LTF	chr3	−9.64493	0.000496
SPRR1B	chr1	8.009959	4.16E-05	PRR4	chr12	−9.34335	0.003043
KRT13	chr17	7.873107	1.42E-05	STATH	chr4	−9.20928	0.001623
CPA4	chr7	7.634678	0.002453	PRR4	chr12	−8.69048	0.001677
KRT4	chr12	7.544229	0.007242	PRR4	chr12	−8.63995	0.001741
KRT13	chr17	7.361434	0.000629	SCGB3A1	chr5	−8.34723	4.29E-06
FAM83A	chr8	7.334105	0.000539	HP	chr16	−7.92955	4.30E-05
SPRR2B	chr1	7.214323	0.000105	PIP	chr7	−7.83139	5.26E-05
SPRR3	chr1	7.085979	0.000475	HPR	chr16	−7.39776	1.51E-05
S100A8	chr1	6.956209	1.50E-05	PI16	chr6	−7.12393	0.000269
SPRR2D	chr1	6.954412	0.000155	PLA2G2A	chr1	−7.12383	0.000317
CLCA4	chr1	6.864341	8.73E-05	CHRM3	chr1	−7.04896	1.40E-05
CLCA2	chr1	6.751895	0.005067	AZGP1	chr7	−6.87092	8.10E-06
PADI1	chr1	6.635014	0.012041	MYH11	chr16	−6.79036	0.000277
S100A9	chr1	6.557596	0.000457	PLA2G2A	chr1	−6.70155	0.00044
SPRR2A	chr1	6.434732	0.000212	CNN1	chr19	−6.68817	7.24E-05
CRNN	chr1	6.350062	0.006995	PPP1R1B	chr17	−6.5309	1.43E-05
RHCG	chr15	6.340818	0.004233	LYZ	chr12	−6.39861	3.31E-05
LYPD3	chr19	6.27005	0.000446	PTGER3	chr1	−6.36724	8.18E-05

### Validation of the Candidate LncRNAs and mRNAs by qRT-PCR

**FIGURE 2:**
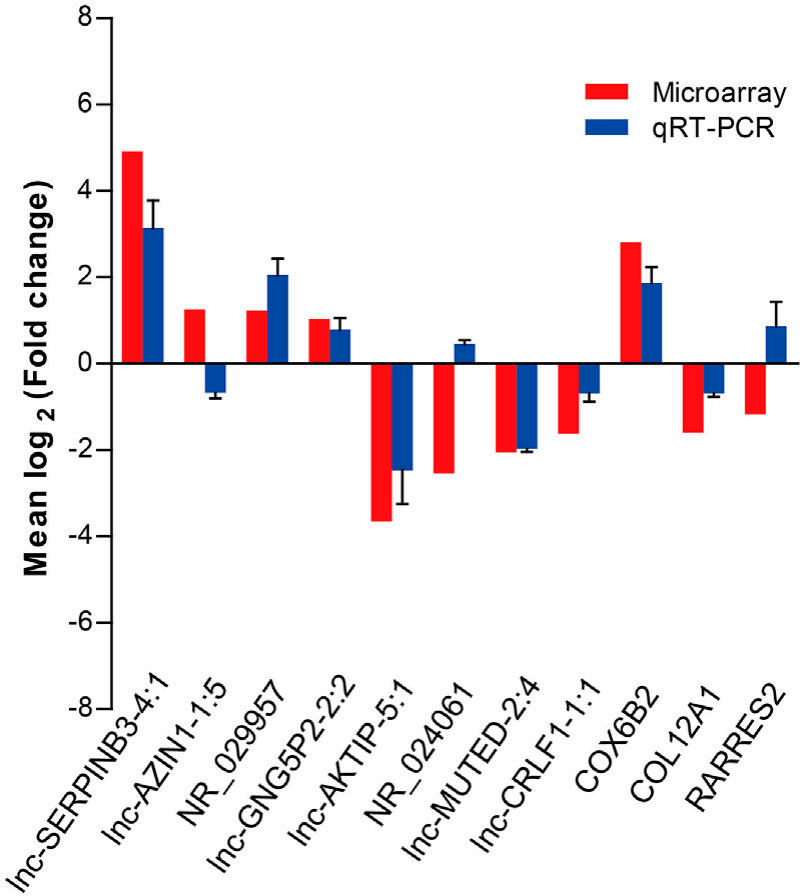
qRT-PCR validation of eight differentially expressed lncRNAs and three mRNAs selected by the microarray assay. Comparison of lncRNA or mRNA expression levels obtained by microarray and qRT-PCR analysis. Upregulated and downregulated lncRNAs or mRNAs are indicated by bars above and below the horizontal axis, respectively. The data from qRT-PCR was shown as mean ± standard deviation (SD).

To validate the microarray data, we performed qRT-PCR to confirm the expression levels of eight lncRNAs and three mRNAs that were randomly selected from the differentially expressed RNAs detected by the microarray experiment. qRT-PCR was performed in two extended panels of the SNIP group (n = 41) and control group (n = 12). The qRT-PCR results for six lncRNAs (lnc-SERPINB3-4:1, NR_029957, lnc-GNG5P2-2:2, lnc-AKTIP-5:1, lnc-MUTED-2:4 and lnc-CRLF1-1:1) and 2 mRNAs (COX6B2 and COL12A1) were consistent with those from the microarray study, while the results for lnc-AZIN1-1:5,NR_024061 and RARRES2 were inconsistent, resulting in a concordance rate of 72.7% (8/11) (Figure 2).

### LncRNA-mRNA Coexpression Network

**FIGURE 3:**
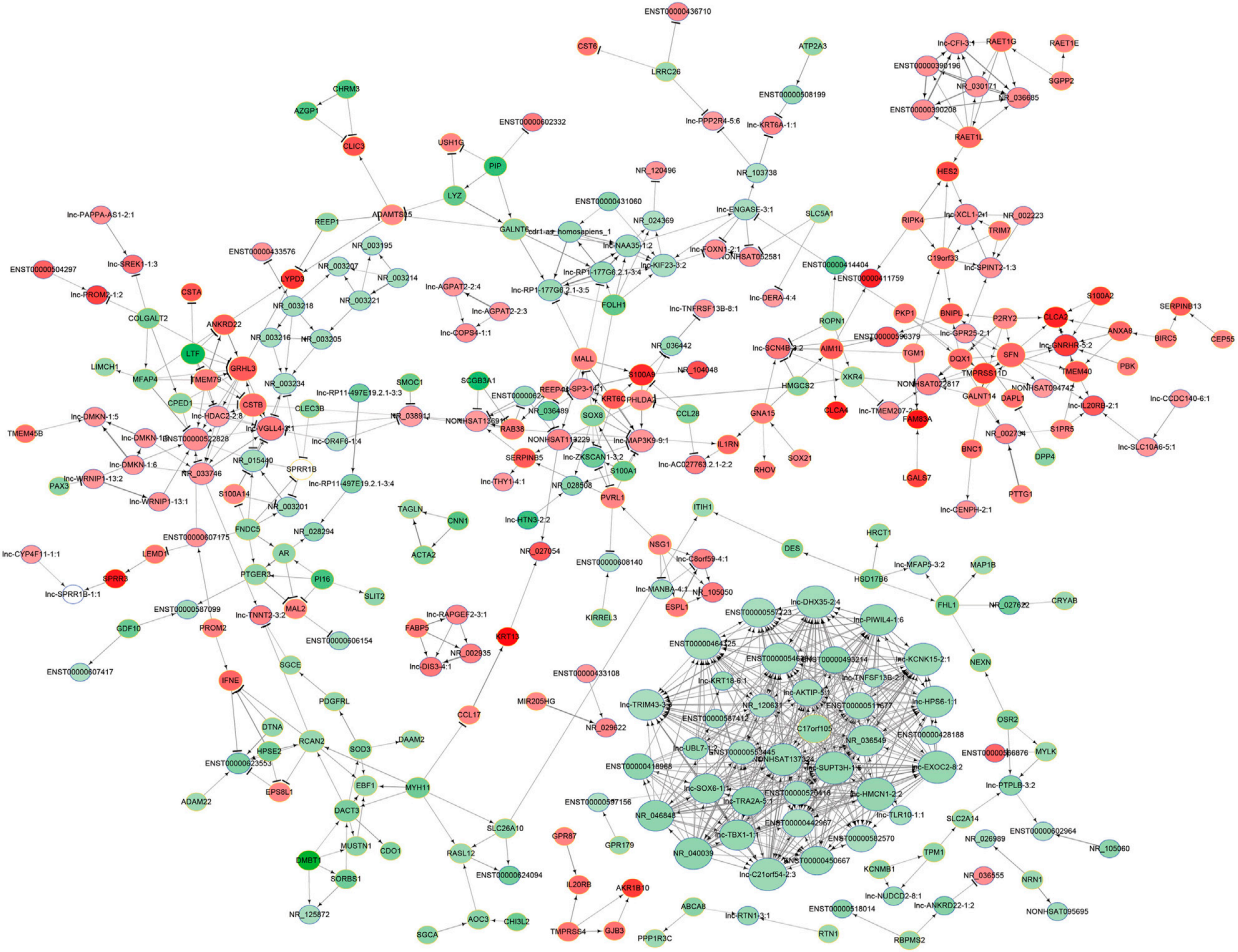
Prediction of the lncRNA-mRNA association network. The coexpression network was composed of 305 network nodes and 769 connections between 155 lncRNAs and 150 coding genes. The red and green circles denote high and low relative expression, respectively. The arrow represents positive regulation, and the flat-head line represents negative regulation.

To explore the potential interaction between the lncRNAs and mRNAs in the SNIP tissues, we analyzed the correlation between the top 400 differentially expressed lncRNAs and mRNAs from our microarray data by calculating R and FDR values. Based on an R value ≥ 0.8 and an FDR <0.005, the lncRNA-mRNA coexpression network was constructed and visualized by using Cytoscape software. The network contained 305 network nodes, including 155 lncRNAs and 150 mRNAs, in which 670 significant correlation pairs were positive and 99 pairs were negative. This network also showed that a single lncRNA could regulate the mRNA expression of multiple coding genes and that some lncRNAs could coregulate the expression of the same gene (Figure 3).

### Enrichment Analysis of mRNAs Coexpressed With LncRNAs

**FIGURE 4:**
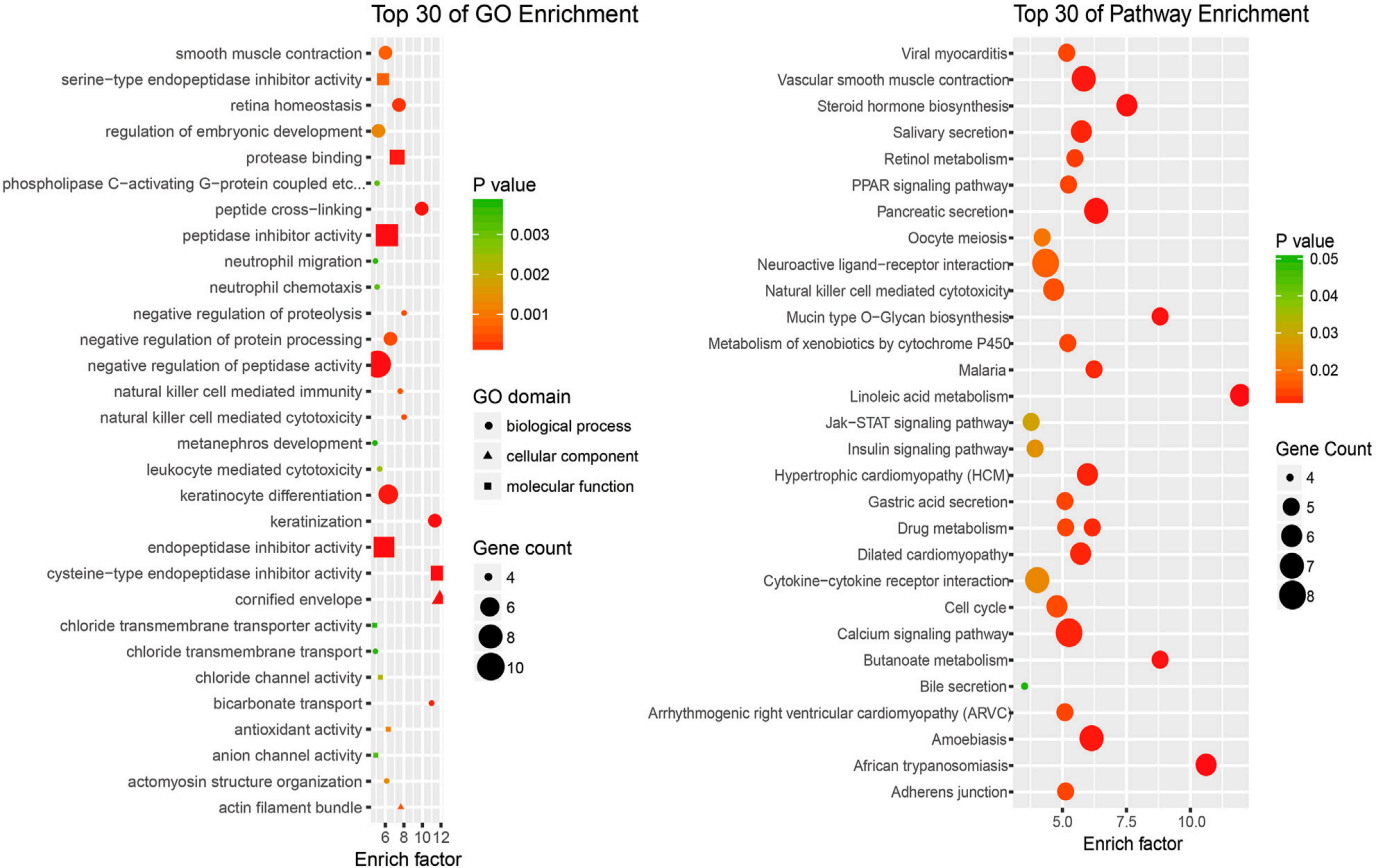
An enrichment analysis of mRNAs coexpressed with lncRNAs **(A)** Top 30 GO enrichment terms **(B)** Top 30 KEGG pathway enrichment terms.

To investigate the potential functions of the differentially expressed lncRNAs in the progression of SNIP, we analyzed the functional (GO and pathway) enrichment of the candidate mRNAs in the lncRNA-mRNA coexpression network. The GO analytical data showed that several significantly overrepresented GO terms were included in the biological process, molecular function and cellular component categories. These mRNAs were enriched in multiple biological processes, such as natural killer cell-mediated cytotoxicity (GO: 0042267), negative regulation of proteolysis (GO: 0045861) and negative regulation of protein processing (GO: 0010955). Pathway enrichment analysis indicated that this subset of differentially expressed mRNAs was involved in the PPAR signaling pathway (ID: hsa03320), Jak-STAT signaling pathway (ID: hsa04630) and insulin signaling pathway (ID: hsa04910) (Figure 4).

### Bioinformatics Analysis of Lnc-AKTIP

**FIGURE 5:**
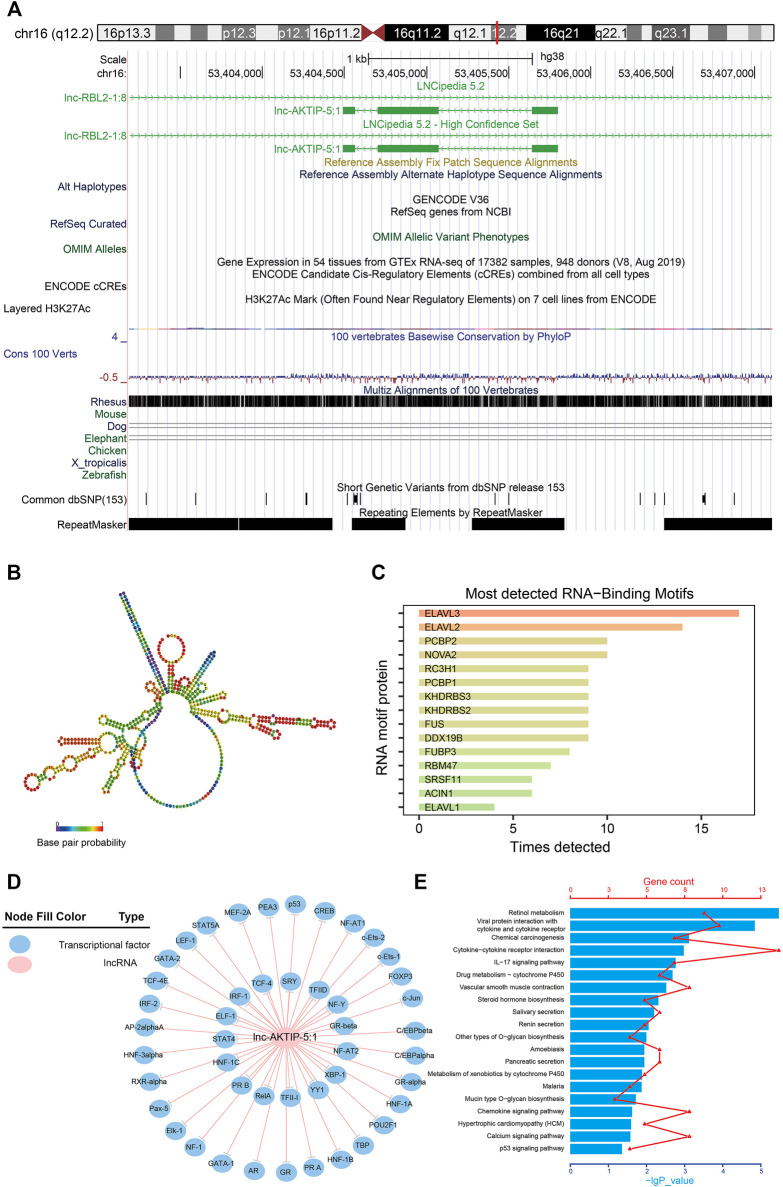
Bioinformatics analysis of lnc-AKTIP **(A)** Chromosome location of lnc-AKTIP **(B)** Optimal secondary structure for lnc-AKTIP **(C)** Interaction between lnc-AKTIP and RNA motif proteins **(D)** Interaction between lnc-AKTIP and transcription factors. Lnc-AKTIP is indicated by a red circle. The transcription factors are indicated by blue circles **(E)** Pathway annotation of the mRNAs coexpressed with lnc-AKTIP.

Lnc-AKTIP was predicted to be found on chromosome 16q12.2 and its DNA sequence was unable to encode proteins (Figure 5A). The optimal secondary structure for lnc-AKTIP had several hairpin loops with a minimum free energy (MFE) of-152.30 kcal/mol (Figure 5B). There were strong interactions of lnc-AKTIP with several proteins. Among these proteins, ELAVL3, ELAVL2 and PCBP2 proteins were found to frequently bind with lnc-AKTIP (Figure 5C). Prediction of potential transcription factors showed that lnc-AKTIP can combine with 46 transcription factors, such as IRF-1, p53, GATA-2, Elk-1, and HNF-1A (Figure 5D). The coexpression network of lnc-AKTIP was involved in the mRNAs of multiple coding genes, such as CXCL8, IL20RB, ABCA12 and RAET1E. These mRNAs were enriched in multiple GO terms, including negative regulation of cell migration (GO:0090051), epidermal cell differentiation (GO:0009913), regulation of epithelial cell proliferation (GO:0050678) and Natural killer cell mediated immunity (GO:0002228) (Table 4). They were also used for further pathway enrichment analysis, and multiple tumor-related signaling pathways, including chemical carcinogenesis, the p53 signaling pathway, and viral protein interactions with cytokines and cytokine receptors, were found to be enriched (Figure 5E; Table 4).

**TABLE 4:** The major mRNAs coexpressed with lnc-AKTIP and their functional enrichment.

mRNAs	Gene ontology (GO) terms	Pathway terms
CXCL8 (NM_000584)	Negative regulation of cell migration (GO:0090051);Epidermal cell differentiation (GO:0009913);Regulation of epithelial cell proliferation (GO:0050678);Natural killer cell mediated immunity (GO:0002228)	Viral protein interaction with cytokine and cytokine receptor (hsa04061);Chemical carcinogenesis (hsa05204) p53 signaling pathway (hsa04115); IL-17 signaling pathway (hsa04657)
IL20RB (NM_144717)		
IL37 (NM_014439)		
ADH7 (NM_000673)		
CYP1B1(NM_000104)		
PMAIP1 (NM_021127)		
SERPINB5 (NM_002639)		
FABP5 (NM_001444)		
HMGCS2 (NM_005518)		
SORBS1 (NM_001290294)		
ALOX15B (NM_001141)		
ABCA12 (NM_173076)		
RAET1E (NM_001243328)		

### Prediction Efficiency of LncRNA as a Potential Biomarker of SNIP

**FIGURE 6:**
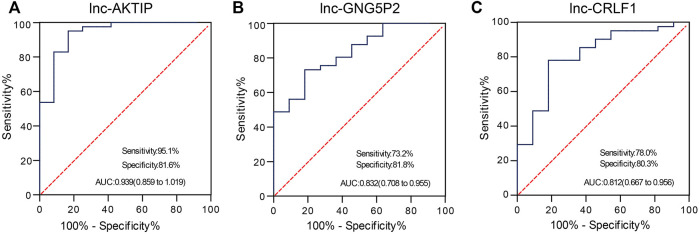
ROC curve analysis was used to evaluate the diagnostic contributions of lnc-AKTIP, lnc-GNG5P2, and lnc-CRLF1 in discriminating SNIP patients. The ROC curve showing the Area under Curve (AUC), sensitivity and specificity of lnc-AKTIP **(A)**, lnc-GNG5P2 **(B)** or lnc-CRLF1 **(C)** as a biomarker of SNIP.

To confirm whether lnc-AKTIP, lnc-GNG5P2 and lnc-CRLF1 could act as diagnostic biomarkers of SNIP, we performed qRT-PCR in two extended panels of the SNIP group (n = 41) and control group (n = 12) and established ROC curve to determine their diagnostic contributions in SNIP. ROC curve analysis revealed that lnc-AKTIP had a high accuracy in distinguishing SNIP patients from controls (AUC = 0.939; 95% CI:0.859–1.019; sensitivity = 95.1%, specificity = 81.6%) (Figure 6A). However, two other lncRNAs were found to not be valuable biomarkers, with low sensitivity of 73.2 and 78%, respectively (Figures 6B,C).

### Relationships Between Clinical Features and Lnc-AKTIP Expression

To determine the potential clinical implications of altered lnc-AKTIP expression, the correlations between lnc-AKTIP expression and clinical features from 41 patients with SNIP were analyzed using the chi-square test. Lnc-AKTIP was revealed to be significantly related to the tumor stage (*p* = 0.007). Among the SNIP patients with upregulation of lnc-AKTIP, 86.4% were found to be in the early staging (Ⅰ+Ⅱ) of SNIP. However, no association was found between lnc-AKTIP expression and other clinical features, such as age, gender, smoking status and tumor recurrence (Table 5).

**TABLE 5:** Relationships between clinical characteristics and lnc-AKTIP expression in patients with SNIP.

Parameter	Lnc-AKTIP		
	Upregulation^a^ (%,N = 22)	Downregulation^b^ (%,N = 19)	*p* Value
Age	—	—	0.994
** **<50 years	8 (36.3)	6 (31.6)	—
** **≥50 years	14 (63.6)	13 (68.4)	—
Gender	—	—	0.699
** **Male	15 (68.2)	14 (73.7)	—
** **Female	7 (31.8)	5 (26.3)	—
Smoking status	—	—	0.921
** **Yes	13 (59.1)	13 (68.4)	—
** **No	9 (40.9)	6 (31.6)	—
Tumor staging^c^	—	—	0.007
** **Ⅰ+Ⅱ	19 (86.4)	9 (47.4)	—
** **Ⅲ+Ⅳ	3 (13.6)	10 (52.6)	—
Recurrence	—	—	0.803
** **Yes	3 (13.6)	3 (15.8)	—
** **No	19 (86.4)	16 (84.2)	—

a Greater than or equal to 0.182 (average relative expression level of lnc-AKTIP, in 41 patients with SNIP).

b Less than 0.182.

c Tumor staging is defined according to a staging system for inverted papilloma described by Krouse, (2000).

## Discussion

The etiology and pathogenesis of SNIP are still unclear and may be related to HPV infection. p53, gelsolin and cathepsin S have been found to be abnormally expressed in SNIP tissue, some of which are possibly associated with the occurrence and development of SNIP (Altavilla et al., 2009; Huang et al., 2010; Cho et al., 2012). However, there are few studies on how to regulate the expression levels of these genes at the posttranscriptional level. Our previous microarray study found that 58 miRNAs, as ncRNAs, were significantly differentially expressed in SNIP tissues, and the expression level of miRNA-214-3p was correlated with SNIP tumor stage and recurrence (Teng et al., 2018). Kakizaki et al. (Kakizaki et al., 2017) also found that miR-296-3p might play a critical role in the malignant transformation of SNIP via the regulation of PTEN and the subsequent inhibition of the PI3K/Akt signaling pathway. To explore the expression levels and potential molecular mechanism of lncRNAs in SNIP, we detected the expression profile of lncRNAs in SNIP tissues by a microarray assay. Our data revealed that a total of 3,435 lncRNAs (1,668 upregulated and 1,767 downregulated) were significantly differentially expressed in the SNIP tissues compared with the corresponding nontumorous tissues. These epigenetic studies suggested that noncoding RNAs likely participate in the occurrence and development of SNIP by regulating the expression of tumor-related genes.

The biological functions of lncRNAs are very complex and have not yet been fully elucidated. It is generally accepted that lncRNAs can regulate the expression level of mRNAs through a variety of molecular mechanisms, such as interfering with the transcription of the promoter regions of protein-encoding genes, inhibiting RNA polymerase II or mediating chromatin remodeling and histone modification to regulate the expression of downstream genes, blocking mRNA cleavage by the complementary double chain structure, and combining with miRNA response elements (MREs) to interfere with the expression of miRNA target genes (Vance and Ponting, 2014; Zhang et al., 2014; Sharma et al., 2021). Thus, lncRNAs can directly bind to transcription factors or change the chromatin structure at the transcriptional level. They can also be involved in regulating mRNA processing and translation at the posttranscriptional level. Our study showed that there was a complex coexpression network between the lncRNAs and mRNAs in SNIP. Furthermore, the mRNAs coexpressed with lnc-AKTIP were enriched in tumor-related biological processes and signaling pathways, such as chemical carcinogenesis, the p53 signaling pathway, and viral protein interactions with cytokines and cytokine receptors. These results revealed that lnc-AKTIP on chromosome 16q12.2 might regulate the pathogenesis of SNIP through mRNAs coexpressed with lncRNAs and a variety of potential molecular mechanisms.

In addition, our bioinformatics analysis indicated that lnc-AKTIP potentially interacted with the PCBP2 protein. PCBP2 is one of the major cellular poly (rC)-binding proteins. Together with PCBP1, this protein also functions as a translational coactivator of poliovirus RNA via a sequence-specific interaction with stem-loop IV of the internal ribosome entry site (IRES), promoting poliovirus RNA replication by binding to its 5′-terminal cloverleaf structure. It has also been implicated in translational control of 15-lipoxygenase mRNA, HPV mRNA, and hepatitis A virus RNA (Sean et al., 2008; Yanatori et al., 2020). Wen D et al. (Wen et al., 2020) reported that LINC02535 functions with PCBP2 to facilitate the repair of DNA damage and then to promote cervical cancer progression by stabilizing RRM1 mRNA. Our study also found that lnc-AKTIP might also bind to multiple tumor-related transcription factors, such as IRF-1, p53 and GATA-2. Thus, lnc-AKTIP may also regulate the occurrence and development of SNIP via some potential interaction mechanisms between ncRNAs and proteins. Therefore, we selected lnc-AKTIP to further analyze its potential clinical implications and diagnosis value.

A growing number of studies have confirmed that lncRNAs can be used as molecular biomarkers for the diagnosis and prognosis evaluation of many diseases. For instance, serum lncRNA LOC284454 has been shown to be a good clinical diagnostic biomarker in nasopharyngeal carcinoma, oral cancer, and thyroid cancer (Fan et al., 2020). Kopczynska M et al. (Kopczynska et al., 2020) reported that in head and neck squamous cell carcinoma (HNSCC) patients, HPV-positive patients with high lncRNA PRINS expression demonstrated significantly better overall survival and disease-free survival than those with low expression. A majority of HPV-positive patients with high PRINS expression demonstrated a high number of immune cells within tumors. It is likely that lncRNA PRINS could be used as a potential prognostic biomarker for HNSCC patients. Along with these clues, we investigated which of the differentially expressed lncRNAs in SNIP tissue could be used as diagnostic biomarkers. Our study demonstrated that lnc-AKTIP could yield a ROC curve area of 0.939 with 95.1% sensitivity and 81.6% specificity in discriminating SNIP patients from controls. Therefore, lnc-AKTIP probably provides great potential as a novel biomarker in the molecular pathological diagnosis of SNIP. However, its specificity needs to be analyzed in depth between SNIP and other sinonasal tumors. In addition, it remains to be further explored whether plasma/serum lnc-AKTIP can serve as a noninvasive diagnostic biomarker for the early detection of SNIP.

The expression levels of lncRNAs are closely related to the pathological characteristics of many tumors. Wang P et al. (Wang et al., 2021) demonstrated that lncRNA NEAT1 might act as an oncogene. Its increased expression was correlated with T grade, neck nodal metastasis, clinical staging, drinking history, and smoking history in laryngeal squamous cell carcinoma patients. We also found that the expression of lnc-AKTIP was significantly associated with the tumor staging of SNIP, which provided a new clue to clarify the epigenetic mechanism of SNIP.

In conclusion, our investigation, although preliminary, has revealed the expression profiles of lncRNAs and their potential biological functions in SNIP. We further demonstrated the potential interaction between lnc-AKTIP and multiple tumor-related transcription factors and the significant correlation between the downregulation of lnc-AKTIP and the SNIP tumor stage. Lnc-AKTIP might act as a putative biomarker in SNIP. Such information would be helpful in further investigating the pathogenesis of SNIP and identifying novel therapeutic targets for the treatment of SNIP patients.

## Data Availability

The original contributions presented in the study are included in the article/Supplementary Material, further inquiries can be directed to the corresponding author.
