# Supplementary material for: Comprehensive Analysis of Sinonasal Inverted Papilloma Expression Profiles Identifies Long Non-Coding RNA AKTIP as a Potential Biomarker
Source: Front Genet. 2022 Feb 2;13:831759. doi: 10.3389/fgene.2022.831759 (PMC8847611; doi:10.3389/fgene.2022.831759)
Supplement: Supplementary file 3 [file Table1.DOCX]

**Supplementary Table 1 Primer sequences used for qRT-PCR**

| **lncRNA/mRNA** | **Forward primer (5' to 3')** | **Reverse primer (5' to 3')** | **Product(bp)** |
| --- | --- | --- | --- |
| lnc-SERPINB3-4:1 | GACATACAGAGTGGGTTGGC | GCTCTGAGGTACAGTGCTGA | 91 |
| lnc-AZIN1-1:5 | ACGGCGAACTTTCTGACCAA | CCCTGGAAGTAGAACAGGGA | 163 |
| NR_029957 | ATGGGCGTCTTACCAGACAT | GCGGATGGACGGTTTTACCA | 71 |
| lnc-GNG5P2-2:2 | CTGCTTTTGCTTCGATGTGCT | GCATGTGGCTTTGGGTAACAA | 112 |
| lnc-AKTIP-5:1 | AGGAACACAGGAAAAGTGGC | GCCTATCAGACACTGCCTTCA | 100 |
| NR_024061 | TGGGAGTTTAGCAGTGGACG | GCGCCTAGCCAAAAAGGTTC | 158 |
| lnc-MUTED-2:4 | TGGACAGCATCAGTCAATGTCA | CGGCTTCAGCAGTTGTTGTC | 187 |
| lnc-CRLF1-1:1 | GAGTGGATTTTTCCGTGGCG | GCACCGCAGTAAGAGGGATT | 90 |
| COX6B2 | AGCCACGAATAATGCCACCA | CCTGGGGTCATGAGTTCACC | 160 |
| COL12A1 | ATCCAGGTTCCGGCTAACAC | TCCTTTGTGATGTCGACCCG | 168 |
| RARRES2 | TGCCCCATAGAGACCCAAGT | GAAGTAGAAGCTGTGGGGGT | 102 |
| GAPDH | GAATGGGCAGCCGTTAGGAA | AAAAGCATCACCCGGAGGAG | 134 |
